# Size Matters: Digital Social Networks and Language Change

**DOI:** 10.3389/frai.2020.00046

**Published:** 2020-07-02

**Authors:** Mikko Laitinen, Masoud Fatemi, Jonas Lundberg

**Affiliations:** ^1^School of Humanities/English, University of Eastern Finland, Kuopio/Joensuu, Finland; ^2^Center for Data Intensive Sciences and Applications, Linnaeus University, Växjö, Sweden

**Keywords:** social networks, Twitter, bot exclusion, data mining, weak ties, social network size

## Abstract

Social networks play a role in language variation and change, and the social network theory has offered a powerful tool in modeling innovation diffusion. Networks are characterized by ties of varying strength which influence how novel information is accessed. It is widely held that weak-ties promote change, whereas strong ties lead to norm-enforcing communities that resist change. However, the model is primarily suited to investigate small ego networks, and its predictive power remains to be tested in large digital networks of mobile individuals. This article revisits the social network model in sociolinguistics and investigates network size as a crucial component in the theory. We specifically concentrate on whether the distinction between weak and strong ties levels in large networks over 100 nodes. The article presents two computational methods that can handle large and messy social media data and render them usable for analyzing networks, thus expanding the empirical and methodological basis from small-scale ethnographic observations. The first method aims to uncover broad quantitative patterns in data and utilizes a cohort-based approach to network size. The second is an algorithm-based approach that uses mutual interaction parameters on Twitter. Our results gained from both methods suggest that network size plays a role, and that the distinction between weak ties and slightly stronger ties levels out once the network size grows beyond roughly 120 nodes. This finding is closely similar to the findings in other fields of the study of social networks and calls for new research avenues in computational sociolinguistics.

## Introduction

This article focuses on social networks and explores network size as a key determinant in the network theory used in sociolinguistics. Building on Granovetter (1973), the theory postulates that individuals form personal communities that provide a meaningful framework for them in their daily life (Milroy and Llamas, 2013). An individual's social network is the sum of relationships contracted with others, and a network may be characterized by ties of varying strength. If ties are strong and multiplex, the network is dense, and individuals are linked through close ties (such as friends). Conversely, ties can be weak in which case individuals are predominantly linked through occasional and insignificant ties (such as acquaintances), and the network is loosely knit. Most importantly, networks contribute to language maintenance and change. Ample empirical evidence shows that loose-knit networks promote innovation diffusion, whereas dense multiplex networks lead to communities that resist change (Milroy and Milroy, 1978, 1985; Milroy, 1987; Lippi-Green, 1989). The underlying reason for the weakness of strong ties in transmitting innovation is the fear of losing one's social standing in a network. Adopting new ideas is socially risky, and we do not want to “rock the boat” in dense social structures.

Even though the social network theory is influential in sociolinguistics, it is mostly based on small data. Most studies have focused on what are usually referred to as ego networks obtained using ethnographic observation. According to Milroy and Milroy (1992, p. 5) this “effectively limits the field of study, generally to something between 30 and 50 individuals.” Moreover, it has been suggested that the quantitative variable of a network “cannot be easily operationalized in situations where the population is socially and/or geographically mobile” (Milroy, 1992, p. 177). In this paper, we concentrate on networks that are larger than small networks of only a few dozen of individuals. This has been done because evidence from social anthropology suggests that average human networks are substantially larger, and individuals can maintain networks with well over 200 nodes (McCarty et al., 2001). Prior empirical work in sociolinguistics has therefore covered only a limited section of possible network sizes.

We have two research foci. First, we test the extent to which social media data from Twitter and computational methods could be utilized to operationalize network ties of highly mobile individuals in very large datasets. Second, we specifically concentrate on the effect of network size on the validity of the theory. We investigate if the fear of losing one's social standing by “rocking the boat” disappears in large strong-tie networks.

To respond to these questions, we discuss two computational methods that can take up large and messy social media data and render them usable for analyzing networks in sociolinguistics, thus expanding the empirical basis from small-scale ethnographic observations. The first method aims at uncovering broad quantitative patterns in data and utilizes what we call a cohort-based method of network size. The second consists of an algorithm-based approach that uses mutual interaction parameters in Twitter and aims to verify the patterns obtained using the cohort-based approach.

By doing so, the article continues our pilot investigation, which suggests that network size is a crucial component in the theory. Our first results indicated that weak ties are meaningful in small networks, but the distinction between truly weak ties and slightly stronger ties levels out when network size increases beyond a certain threshold level (Laitinen et al., 2017). This pilot was based on social media data that had not yet been cleaned of unwanted software robot data (i.e., bots). In the present study, we attempt to replicate the study using a more accurate dataset from which we have removed bots by means of machine-learning techniques and by using novel computational methods to test our first observations. Bot content can result in inaccuracies, and previous computational sociolinguistic studies rely on a range of methods when bots are handled. Their presence may be recognized, but they are nevertheless included in the results (Huang et al., 2016; Laitinen et al., 2017). Other methods, such as excluding material by using metadata parameters, are occasionally used (Coats, 2017), but as we demonstrate below in section Material and Methods, more advanced solutions are available.

As shown in the next section, the role of network size in sociolinguistics is an understudied phenomenon, which not only requires new tools but could also shed light on the contrast between strong and weak ties in innovation diffusion. One example is that while the weak-tie model is beneficial, it has recently seen substantial theoretical elaboration, and recent advances have broadened the understanding of networks ties as a unidimensional concept (Aral and Van Alstyne, 2011). What is clear is that weak-tie and close-knit networks are different for small ego networks obtained through ethnographic methods, but if network size is ignored, the social network theory is not fully consistent with some of the major findings in sociolinguistics. First, it is widely held that there is one period when individuals maintain maximally close ties with their peers, and that is adolescence (Chambers, 2003, p. 90–91). Yet, the role of adolescents in language change is indisputable and verified in both real-time and apparent-time studies of change in progress (Labov, 2001, p. 76; Tagliamonte and D'Arcy, 2009). There might, of course, be other reasons than interpersonal ties during adolescence that lead teens to diverge from adult norms, but network size deserves to be studied in more detail. Moreover, ample macro-level evidence suggests that densely populated and sufficiently large working-class urban areas have, throughout history, been sites for innovations (e.g., the Jewish quarters all over Europe, Harlem in New York City, or St. John's Ward in Toronto). Pan et al. (2013) suggest that it is the size and density of the ties of a center that are crucial for information diffusion. They investigate social-tie density and information contagion in urban populations, and their quantitative model shows how density, with both weak and strong ties, drives the “super-linear” growth of interaction and information diffusion. Close-knit urban centers may, of course, be sufficiently large to sustain individuals with weak ties through whom innovations spread to a community, but we simply do not yet know whether the role of weak and strong ties levels out beyond a certain threshold level.

Section Social Networks in Variationist Sociolinguistics Discusses not only the theoretical basis of social networks in sociolinguistics but also reviews recent insight from complex systems analysis and social network theory. Section Material and Methods details the material and the two methodologies. Section Results presents the results, and, finally, section Conclusions discusses the implications of our findings.

## Social Networks in Variationist Sociolinguistics

Social network analysis in the variationist paradigm transpires from the idea that individuals establish interpersonal ties of varying strengths to form communities. These personal social networks are not independent from other socio-cultural frameworks but are closely related to other variables, such as gender and social layer (Milroy and Milroy, 1992). Interpersonal ties influence the rate at which innovations are adopted and how they diffuse into a community. Sociolinguists have shown that strong networks tend to maintain and support local norms and provide resistance to the adoption of competing norms from the outside. Conversely, conditions that are characterized by weak and uniplex ties are important channels for outside influence as people in such situations tend to accommodate to each other linguistically. Contact situations with weak ties therefore contribute positively to the spread of innovations.

This finding builds on Granovetter's (1973, p. 1365) observation that “only weak ties may be local bridges.” More people can be reached through weak ties, but not all weak ties serve this function, “only those acting as bridges between network segments” (1983, p. 229). To explain this somewhat counterintuitive observation, Granovetter (1973, 1983) argues that close-knit networks encourage local cohesion and norm-enforcing communities where the adoption of innovations is risky. Loose-knit networks with individuals already on the social fringes are more susceptible to external innovations. In addition, weak ties may be expected to be more numerous among mobile individuals and are thus more likely to contribute to the diffusion of an innovation.

In variationist sociolinguistics, network ties have been operationalized in various ways (Milroy and Llamas, 2013). In the Belfast study, they were measured using five indicators to establish how complex and dense a particular tie was. The indicators consisted of (a) having membership in a locally-based group, (b) having ties with at least two households in the neighborhood, (c) sharing a workplace with two or more individuals from the neighborhood, (d) sharing a workplace with same-sex individuals from the neighborhood, and (e) being involved in voluntary activities with individuals from the same workplace. The responses resulted in a network strength scale, which formed an independent variable, and these values were compared to the dependent (phonological) variables. The results show that the individuals with strong network ties with the local community also exhibited the highest share of local, vernacular speech, and “that a close-knit network has an intrinsic capacity to function as a norm-enforcement mechanism, to the extent that it operates in opposition to larger scale institutional standardizing pressures” (Milroy and Milroy, 1985, p. 359).

A large body of variationist sociolinguistic literature exists in which the network-based approach has been applied to small contemporary communities (Milroy and Llamas, 2013). Milroy and Milroy (1978) use 46 speakers from three urban, blue-collar Belfast communities, and the network ties were established through a participant observation process in which a researcher was introduced to a community by means of a friend-of-a-friend technique. Of these, 12 had network scores qualifying them as weak tie individuals. The same also applies to Granovetter's (1973, p. 1368–1371) study as his empirical data came from a random sample of 100 personal interviews taken from the total sample of 282. Carefully constructed personal networks are obviously important, but the availability of social media data also forces us to ask if the model holds when tested with considerably larger networks.

Network size has not been considered as a separate independent variable in variationist sociolinguistics (Milroy and Llamas, 2013). The model has been applied to large communities in macro-level approaches (Milroy and Milroy, 1985; contrasting Icelandic and English; Raumolin-Brunberg, 1996; investigating mobility as a result of the Civil War in the seventeenth-century England, and Nevalainen, 2000; examining patterns of mobility in Early Modern London). However, while all of these studies are rich in linguistic evidence, they nevertheless contain no direct quantitative evidence of how much weak ties actually increase in the settings that are examined. They rely on indirect evidence of migration patterns, population growth and birth/death rates for instance, but information of average network size per community is not detailed.

Recent findings in social anthropology have shown that an average network size is larger than a few dozen individuals. Dunbar (1992, p. 469) has suggested that the neocortex size and the number of neocortical neurons impose a cognitive upper limit on an individual's information-processing capacity. These limit “the number of relationships that an individual can monitor simultaneously” to around 150 nodes. Additionally, McCarty et al. (2001) use two methods to estimate the size of average networks. They use what they term the scale-up and summation methods, and the results show “a remarkable similarity between the average network size[s] generated by both methods (~291)” (2001, p. 28). They estimate, however, that network sizes for various subpopulations can be substantially larger. These include clergy, politicians, labor organizers, and diplomats.

**Figure 1:**
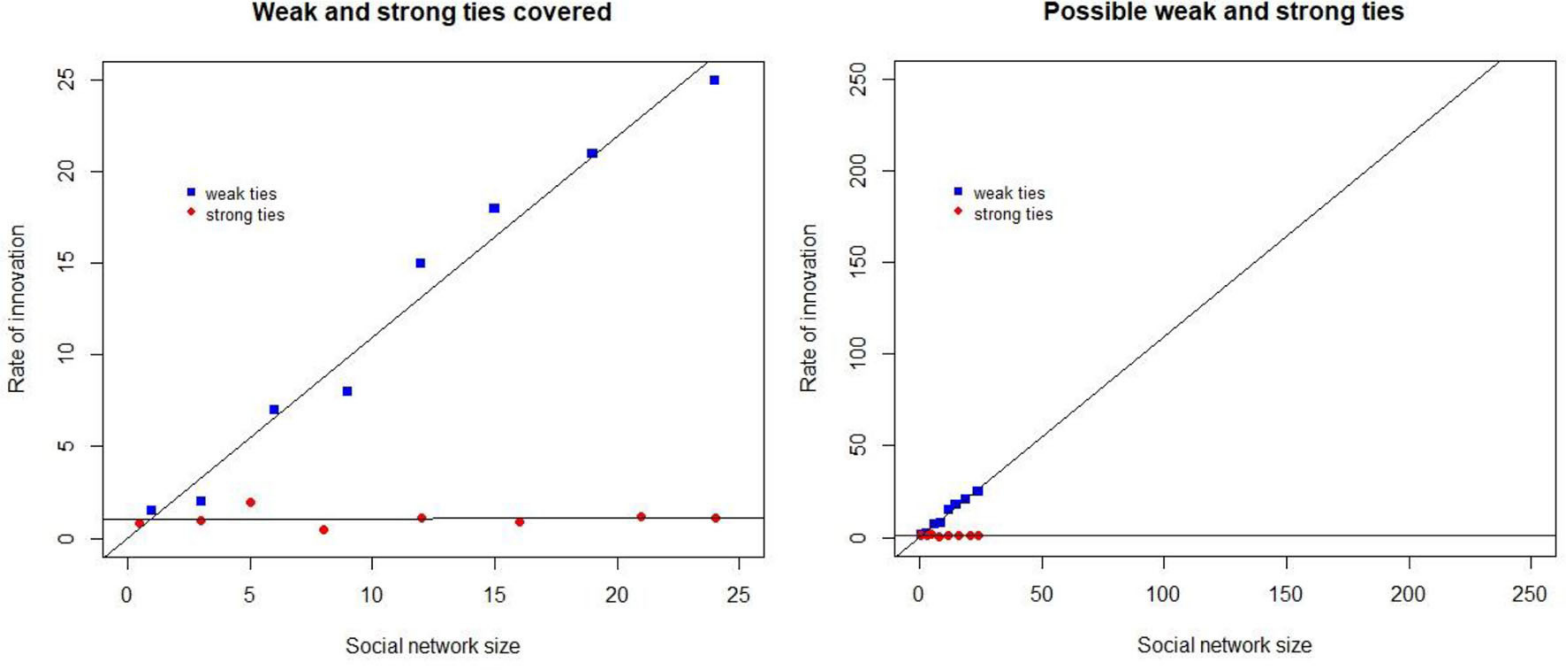
A schematic representation of the network sizes covered (**Left**) and also the cognitively possible networks (**Right**).

Sociolinguistic research has covered a part of the feasible network sizes. Figure 1 visualizes this with the aid of dummy data. The x-axis indicates the size of networks and the y-axis the rate of innovation adoption for network types. The left-hand part shows the size of the networks covered, while the right shows how these fare with cognitively possible human network sizes.

We added a regression line to the visualizations but given the absence of empirical evidence it is impossible to know whether the line continues if we have evidence exclusively from small networks.

Recent findings from fields outside sociolinguistics suggest that network sizes play a more substantial role than previously thought. Ma et al. (2019) focus on trust in public and private social media groups, surveying 6,383 Facebook Groups users. Their observations show that people trust private groups more than they do public groups, which is to be expected. However, the differences between group types disappear once the group size exceeds circa 150 members. When networks become larger, individuals are no longer be able to perform the mental reasoning of who actually is in the group and who is not. Therefore, the difference between network types levels in large networks.

Moreover, increasing empirical evidence has recently led social network scholars to question the unidimensionality of the weak-tie model. Brashears and Quintane (2018) for instance elaborate on the idea of bandwidth in social contacts as an additional dimension. This concept refers to the total flow of information and accounts for capacity, frequency, and redundancy of network ties. Their model shows that even though humans acquire a smaller proportion of new ideas through strong contacts, the greater bandwidth of these contacts means that more total content is transmitted through these contacts. Strong contacts could therefore be more likely to transmit a greater share of novel information than weak ties, which could explain the role of large urban working-class centers as places for innovation.

We investigate networks in Twitter and operationalize them using metadata available for each account. These are related to network size and mutual interaction patterns. Previous studies in computational sociolinguistics have used such information more to extract social network structures (Nguyen et al., 2016), but less to deepen understanding of the social network theory, which is the locus of this article. Eleta and Golbeck (2014) apply machine learning to study how social network characteristics and linguistic profiles influence language choice and how multilingual users of Twitter mediate between language groups in their social networks Their data consist of 92 ego networks, and the observations show that the proportion of English users in the network is the most significant predictor of language choice. Moreover, if a network consists of L2 users, this will increase the likelihood of L2 use. Kim et al. (2014) investigate how virtual networks impact multilingual practices, and they quantify “the degree to which users are the ‘bridge-builders’ between monolingual language groups.” Hale (2014) studies networks utilizing mentions and retweets, and his results confirm the central role of multilingual users, and those who use English in particular, as the bridging forces in the network.

## Materials and Methods

To test the computational methods, we use two sets of Twitter data. Section A Cohort-Based Approach to Network Size uses evidence from the *Nordic Tweet Stream* corpus (NTS), which is a real-time monitor corpus of geolocated tweets and their metadata from the five Nordic nations (Laitinen et al., 2018). Section An Algorithmic Approach to Networks in Sociolinguistics utilizes an algorithm-based method, which makes use of mutual interaction data from a set of accounts from the Nordic region.

The NTS is being collected using the free Twitter Streaming API and the HBC (https://github.com/twitter/hbc) as the downloading mechanism. We apply a double filtering with the geolocation information and the Nordic country codes to ensure that the material originates from the region (Laitinen et al., 2018). While tweet data offer an efficient way of capturing big societal data, there are limitations. As an illustration, users who do not want to share their geolocation are not included. Depending on privacy settings and the geolocation method used, tweets either have (a) an exact location specified as a pair of latitude and longitude coordinates or (b) an approximate location specified as a rectangular bounding box. These geolocation data are available in the metadata attached to the message. Alternatively, no location at all is specified. For location, the data are derived either from the user's device itself (using the GPS) or by detecting the location of the user's Internet Protocol (IP) address (GeoIP). Exact coordinates are almost certainly from devices with built-in GPS receivers (e.g., phones and tablets). The GeoIP-based device location can be tricked by using proxy gateways. Attempting to hide one's location is probably most common amongst users with a malicious intent, such as bots.

To exclude bots and to increase data accuracy, we use a machine-learning algorithm developed by Lundberg et al. (2019). The version recognizes automatically generated tweets (AGTs) written in English and in Swedish. We define an AGT as a tweet in which all or parts of the natural language content are generated automatically by a bot or other type of program. The algorithm makes use of nine numerical and nominal properties that can be computed directly from the tweet metadata. The accuracy rate of the algorithm is over 97%. The results in section A Cohort-Based Approach to Network Size exclude possible bot accounts, whose share of AGTs is >50%, and section An Algorithmic Approach to Networks in Sociolinguistics focuses on genuine human accounts that have been selected manually.

The first method (based on cohorts) does not assume a pre-existing social network as the starting point but rather aims at uncovering quantitative patterns in the data. To measure network sizes and to correlate size with the rate of innovation, we use two metadata attributes available for each tweet. They measure the number of one's online friends and followers, and networks are operationalized as follows: The number of followers indexes truly weak ties (i.e., requires no action from a user), and the number of friends is an indication of slightly stronger links (i.e., requires user effort). We suggested previously that these metadata offer a way of measuring social networks and are ideal for research purposes, because they are automatically generated and hence they reduce the observer bias (Laitinen et al., 2017). They are also freely available to researchers with intermediate computing skills.

Similar to Milroy (1987), we operate under the assumption that social networks are abstractions, but we also propose that information from digital social network applications can be used to distinguish between ties of varying strengths. Friend and follower counts are useful indicators of social networks because of their differing qualities. Our definition of truly weak ties and slightly stronger ties is similar to Granovetter's (1973, p. 1361) assumption that the “strength of a tie is a (probably linear) combination of the amount of time, the emotional intensity, the intimacy (mutual confiding), and the reciprocal services which characterize the tie.” His methodology assumed stronger ties to be “friends,” while weak ties consisted of “acquaintances,” very similar to what we do below. By the same token, while we do not claim that friend count would indicate stronger ties in the sense in Milroy (1987), we assume that our operationalization of digital social networks is closely similar to the underlying idea of networks. Indeed, Milroy (1992, p. 178) argues that “a tie is ‘weak’ if it is less strong than the other ties against which it is measured,” which also holds true for the follower counts when compared with friends.

The second method, the algorithm-based approach, zooms in on a set of real networks extracted by accessing account information through the Twitter API. We employ data such as friends and follower patterns, re-tweets, mentions, and directed messages. The accounts are anonymized, and we work with two types of network.

- Large (100–300 nodes) weak-tie networks
- Large (100–300 nodes) close-tie networks

We identified a set of accounts similar user profiles and extracted all interaction data available. The policy limitation of the API allows accessing up to 3,200 of the latest messages for each unprotected account. The account holders are from the metropolitan areas of Helsinki and Stockholm, are not working in academia, identify as males, have >10 messages primarily in English, and have more than 300 friends and followers. The last figure comes from a study that estimates median network sizes for multilingual individuals (Laitinen and Lundberg, 2020).

We narrowed the candidate accounts to ten and extracted their networks, including recent tweets and mutual interaction profiles. We excluded verified accounts (i.e., subpopulations with anomalous networks of politicians/celebrities/businesses) and accounts with more than 1,500 contacts (friends + followers). This was done to ease the time required for extracting mutual interaction data from large social networks. It is important to note that, while the number of accounts is small, the data extraction through the API takes circa 3–6 h per account (Table 1).

**Table 1:** Raw statistics for the data used in section An Algorithmic Approach to Networks in Sociolinguistics.

**Account**	**Friends**	**Net size**	**Loss rate (%)**	**Tweets**	**Retrieval (in mins)**	**Text collection (in mins)**
account_01	409	221	46	312,350	230	38
account_02	335	166	51	253,758	181	33
account_03	309	195	37	286,945	201	33
account_04	332	175	47	150,774	184	25
account_05	201	105	48	100,915	105	14
account_06	418	132	68	192,944	140	23
account_07	468	281	40	316,944	291	41
account_08	448	286	36	322,566	303	40
account_09	418	216	48	189,628	229	26
account_10	496	297	40	516,686	282	67

Even though the algorithm-based approach is tested with ten accounts, the size of our data is large. For instance, the mean network size is over 200 individuals (207), and the size of the textual data is over 2.6 million messages. In Table 1, the net size represents the number of collected accounts for the network (number of nodes in the graph). The loss ratio indicates the percentage of accounts lost after filtering.

The mutual interaction patterns are subjected to algorithms in order to assign labels of weak or strong networks to the accounts. The algorithms are explained in detail below, but they are mainly from the graph theory and the set theory, and some of them have been developed by us. For instance, we use betweenness centrality, which is a measure based on finding the shortest path between nodes (Freeman, 1977; Brandes, 2001) and closeness centrality (Perez and Germon, 2016). Kuikka (2018) argues that betweenness measures identify nodes that act as brokers between communities and are used to detect the density of how people are connected to each other in a network. We also use Jaccard Similarity Coefficient (JSC), which is a symmetric measure that calculates the similarity between two sets, and it is used to measure the similarity between accounts in terms of the number of common followers/friends. The assumption is that the share of common friends/followers is higher in a strong-tie network than in weak-tie settings. In addition, we assign weights to each account in the network and employ a method which we call disjointness. This last method enables us to estimate how well the nodes in a network are connected if the ego node were to be removed. The network labels are therefore multidimensional.

As for the dependent variables, we employ items that are frequent enough to be used in the testing phase. First, the cohort-based method uses the dominant language for each account. This information is available in the NTS metadata, and the share of English messages per account is correlated with network sizes. As our data come from the Nordic region, it ought to be noted that while English has no *de jure* position in the region, it is increasingly used as a lingua franca. Space does not permit us to discuss the sociolinguistic diversity of the region, but see country overviews in Modiano (2003), Preisler (2003), Leppänen et al. (2011), and Graedler (2014). Previous studies that use Twitter data have suggested that a great majority of messages in one location, a region for instance, are from residents of that location (Gonçalves et al., 2018; Lamanna et al., 2018) and not from visitors and tourists. The cohort-based method uses information from tens of thousands of accounts, and we assume that our dataset is reliable, given the general limitations of Twitter data. We use automatically-assigned language labels, and although automated language identification methods are not entire accurate, the agreement between human coders and Twitter's language recognition system is fairly high for languages written in the Latin alphabet (Graham et al., 2013).

Second, the algorithmic approach uses a mixture of linguistic features available in the tweet text. These features consist of contracted forms (*won't*, ‘*ll, I'm* etc.), and *NEED* to used as a semi-modal auxiliary. These features are qualitatively different as the contracted forms index colloquial, spoken-like use (Biber et al., 1999, p. 1128–1132), while *NEED to* is currently undergoing change in English (Leech, 2013) and is highly pervasive in ELF use in the Nordic region (Laitinen, 2016).

## Results

### A Cohort-Based Approach to Network Size

We illustrate the cohort-based method first using data from 199,832 accounts from the NTS, from which we removed subpopulations with anomalous network profiles, as defined in section Social Networks in Variationist Sociolinguistics. After the initial results, we test the findings with data from which software bots are removed. These bot-free data consist of 90,887 accounts, obtained from the NTS but limited to Sweden only (labeled as NTS-Human-Swe).

The null hypothesis is that increasing the number of network ties does not lead to increases in the share of English per account. The cohort-based approach for both categories is specified in (1)–(6) (it refers to followers in the NTS, but the same procedure applies to friends and to both datasets):

(1) We sort out all the accounts based on their followers' counts.
(2) The accounts are divided into N equally-sized cohorts where cohort 1 is the 199,832/N, and it has the lowest follower count, and cohort N has the highest. N can of course be adjusted.
(3) We compute the percentage of tweets written in English per each account.
(4) The language identifier used is Twitter's own language identification tool, the accuracy of which is discussed in section Material and Methods.
(5) We can adjust the proportions of English in the tweet stream (EngMajor) for each cohort and associate the cohorts with the EngMajor percentage. The results here use >50% share of messages in English (for other proportions, see Laitinen et al., 2017).
(6) We correlate the cohorts against the percentages and visualize them.

**Figure 2:**
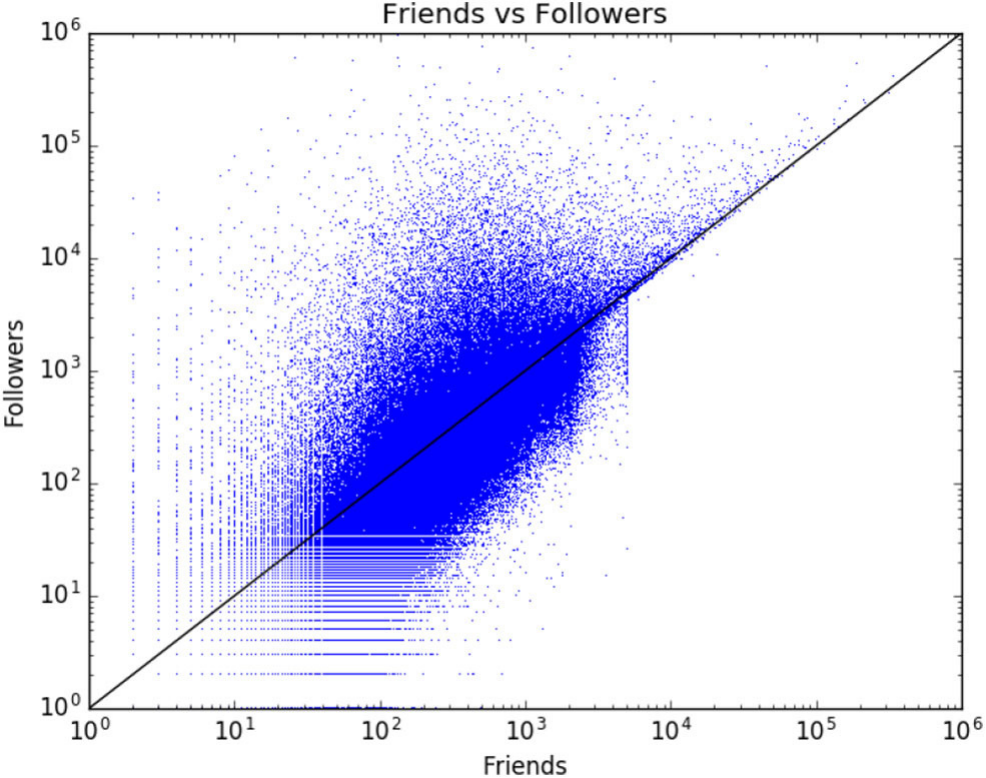
Friends and followers visualized (199,832 accounts).

An average account profile in the NTS is such that the median size of networks is 235 friends and 195 followers. Figure 2 shows how the friend and follower counts are distributed in the data. There is a relatively straightforward (x = y) spread of the values. The only exception is the friends category, in which Twitter imposes an upper limit of 5,000 friends that each individual account can follow (https://support.twitter.com/articles/66885#). The only way to increase one's friends count is to gain new followers, and therefore there is an even more direct correlation of friends/followers after the 5,000 mark.

**Figure 3:**
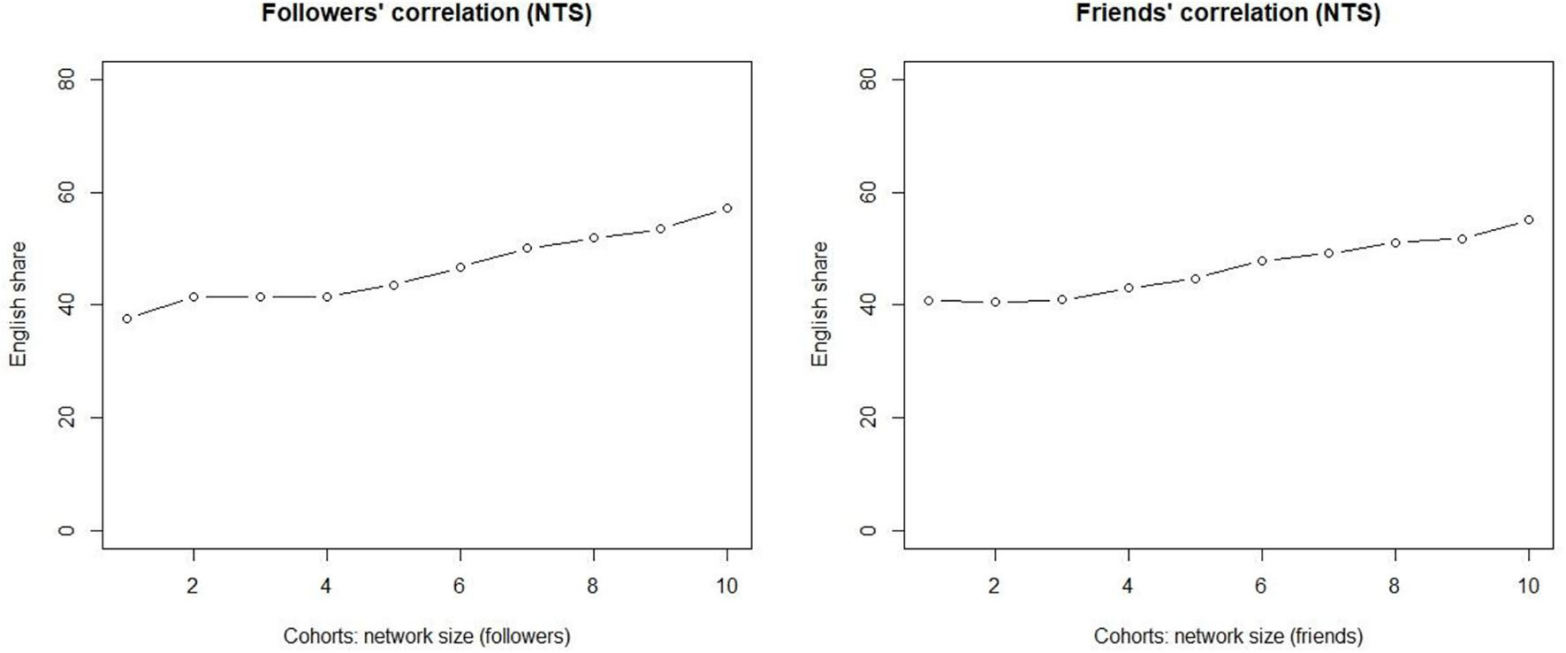
The correlation between followers (**Left**) and friends (**Right**) and the share of accounts in which English dominates.

Figure 3 (left) illustrates a 10-cohort division visualizing how cohorts differ in terms of the >50% percent threshold. The result shows that more Twitter followers means more messages in English, with the non-parametric Kendall tau correlation coefficient (0.956) indicating a strong positive correlation between the two vectors at statistically significant levels (*p* < 0.0001). Note that cohorts 1–4 are accounts with fewer than the median number of followers (i.e., 195).

The quantitative pattern with these truly weak ties is clear. The correlation between the follower counts and the use of English is linear, and the correlation is strong. Of the accounts in which the number of followers is lower than the median, roughly 40% have the majority of their messages in English. The higher that we move in the cohorts, the higher is the share of such accounts. At the other extreme, in the cohorts with the highest number of followers over half of the accounts fulfill the criterion.

The quantitative pattern for the slightly stronger ties (friends) is shown on the right. The correlation between the number of friends and the increase in the use of English is strongly positive, with the Kendall tau correlation coefficient at 0.867 (*p* < 0.0001), i.e., for all of the 199,832 accounts in the dataset, more online friends means a larger share of messages in English.

However, contrary to what is observed with truly weak ties, the stronger network index behaves differently. For small networks, the increase in network size has no impact on the response variable. It is only from cohort 4 onwards that the share of EngMajor increases when we increase the number of friends in the network.

These results suggest that there is a straightforward correlation in the truly weak tie networks, but the friend data indicates that the distinction between weak ties and stronger ties levels out when the network size is large enough. If we had restricted our analysis only to traditional small networks of 30–50 nodes in ethnographic attempts, our data would have confirmed the customary finding related to the diffusion of innovations and network strength. That is, weak ties promote change, and stronger ties prevent it. However, the results obtained using this approach suggest that this is not necessarily the case. Once the network size grows to become large, the traditional distinction between weak and stronger ties disappears. Note that we are not referring to the percentages of the accounts, but to correlational patterns of the variable. Large networks here mean that the network sizes are still within the cognitive limits (see section Material and Methods).

To explain this finding, we must balance between the limitations and the advantages of our data. The most obvious limitation is that we might observe a random quantitative pattern that emerges from messy data. Moreover, we do not know anything about the density or the multiplexity of the network ties but can only assume that the friends category represents a slightly stronger network index, since it involves an active decision to follow someone. The friends network index might also include a greater share of interactive networks. To tackle the limitations, the next section applies a different method and approaches ego networks.

The obvious advantage is the size of our data. Each cohort in Figure 3 consists of nearly 20,000 accounts, and we are not restricted to small ethnographic records. The network size for the first three cohorts is 0–122. As pointed out earlier, the median number of friends is 235. The results support rejecting the null hypothesis, but the threshold level of 122 stems from an arbitrary value of ten cohorts.

**Figure 4:**
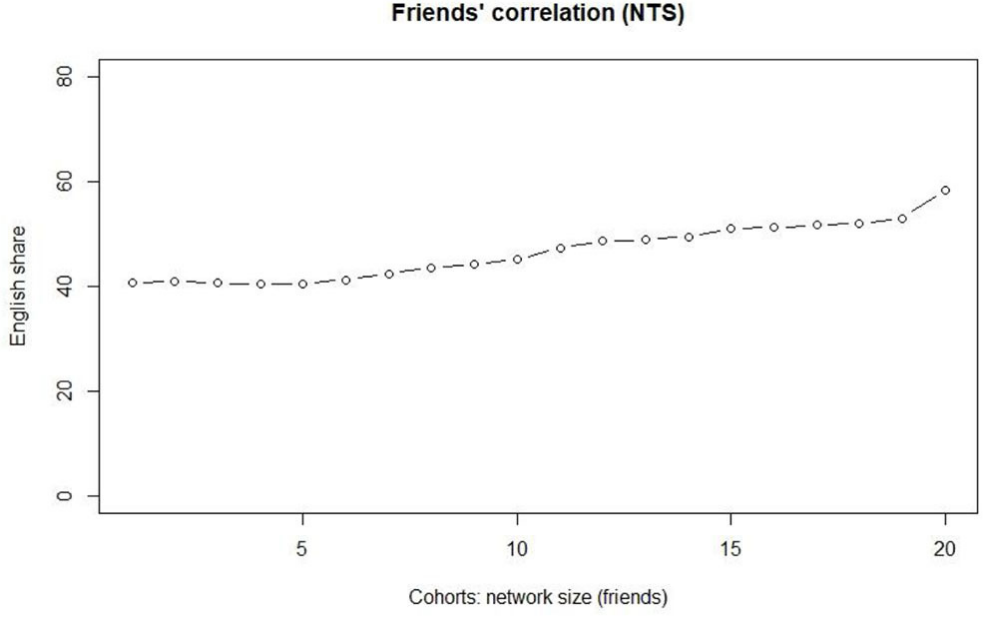
The correlation between 20-cohort friend category and the EngMajor.

Figure 4 tests the observations using 20 cohorts. As the interest is on slightly stronger ties, we only use the friends data. The figure confirms the observation and indicates a leveled proportion of EngMajor for the first five cohorts. After that, the network size correlates positively with the increasing use of English in the tweet stream. The Kendall tau correlation coefficient is 0.905 at a statistically highly significant level (*p* < 0.0001).

Cohorts 1–5 consist of networks of <100 individuals, and a marked increase takes place only after cohort 5 (100–122 individuals). The share of accounts with a >50%+ share of tweets in English increases systematically for each cohort so that for cohort 6 it is 41.2%, and for cohort 19 it is 51.9%. Cohort 20 has its friends count at over 1700, and according to our present understanding, these represent “evangelists” in the Krishnamurthy et al. (2008) sense, i.e., they are more or less automated bots aiming at increasing their friends basis automatically.

Figure 4 suggests that the threshold network size after which the distinction between weak ties and slightly stronger ties levels is of around 122 nodes. Next, we zoom into the bot-free data, and the main question is whether we can replicate the findings using the bot-free data. Overall, the number of bots in the Swedish subset is low (1,149 accounts = 1.0%), but they generate a high number of tweets (404,804 = 7.6%). The majority language in the bots is English, since nearly 20% of all of the English tweets were identified as AGTs, but the corresponding share for Swedish was <2% (see Laitinen and Lundberg, 2020). The visualizations also exclude the smallest networks of fewer than five nodes.

**Figure 5:**
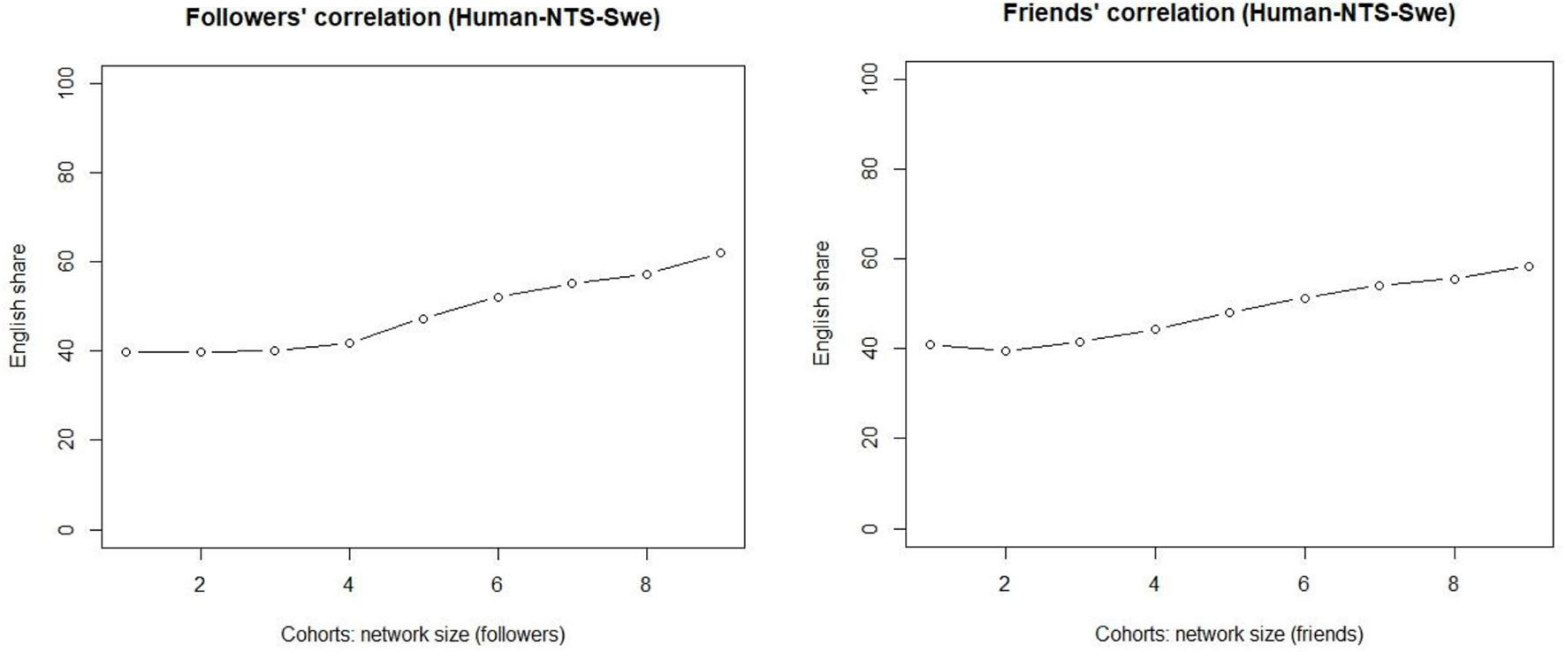
Bot-free correlations of truly weak ties (**Left**) and slightly stronger ties (**Right**).

The bot-free quantitative patterns are shown in Figure 5, and they are similar to those observed earlier. As for followers (left), they show a linear increase in the share of messages in the English per cohort as we move to the right on the x-axis. The correlation between network size and the share of English is not only straightforward but also statistically significant, as the Kendal tau correlation coefficient is one (*p* < 0.0001). For smaller networks, the share of English is around 40%, and it increases for every increase in the network size, so that the share for the largest networks is well over 50%. The increases are slight, but the shares of the English use nevertheless increase for each cohort. Once the network size grows larger, we observe more noteworthy increases.

The right-hand side visualizes the slightly stronger ties (friends) and verifies the initial observations. These results confirm the findings presented above. The observations show that the correlation with slightly stronger ties is equally linear, and this is also supported by the Kendal tau value (0.944, *p* < 0.0001). However, the share of English actually decreases for the small stronger-tie networks. That is, the empirical evidence presented here suggests that truly weak ties and slightly stronger ties behave slightly differently for small networks, but the distinction disappears once the network size grows larger. The share of English remains flat for cohorts 1–3 of the truly weak ties (left), while the share actually decreases for the slightly stronger ties for the smallest networks (right). Cohort 4 consists of those whose network size exceeds 120 nodes.

The present section has presented our cohort-based approach to measuring networks in social media. While we acknowledge that the method is straightforward, it has obvious benefits for this type of big and rich data approaches to language variability and social networks. The method is light in terms of computing power, as the values can be easily obtained from the data stream. In addition, we can use data in their entirety since each account makes the values directly available with minimal or no data loss.

The obvious difference between this approach and the ethnographically-oriented data-collection in Milroy (1987) is that our method does not deal with ego networks but rather takes a top-down approach, correlating network size and a linguistic feature. As for the innovation, previous studies have shown that English in the Nordic region is closely associated with age; this means that the younger generations clearly use English as an additional tool more often than do the older groups (Leppänen et al., 2011). Unfortunately, age is not included in the metadata parameters in the raw data, and its role cannot be controlled.

The main finding here is that we can confirm our pilot results in Laitinen et al. (2017). The new cohort-based findings using bot-free data suggest that network size plays a role in leveling the differences beyond a certain threshold. The following section will turn its attention to ego networks.

### An Algorithmic Approach to Networks in Sociolinguistics

**Figure 6:**
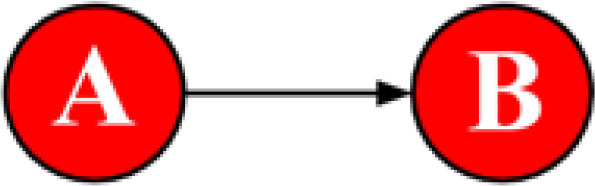
A simplified example of a directed graph.

This section digs deeper into digital networks and uses an algorithmic method that complements the results above and provides tools for analyzing networks of mobile individuals. We operate with the 1-step neighborhood, which consists of a focal node, ego, and nodes directly connected to it. We also include the connections between nodes (degree 1.5). Twitter is a directed*-*graph network, and we are interested in what accounts “see” instead of how they are “seen,” and consider friends rather than followers in the analysis. Consequently, we deal with a graph-based structure in which nodes represent accounts and directed edges are considered as a friend relationship, as in Figure 6, which visualizes two nodes in which A is either following B, or B is a friend of A.

The method assumes that account activities and mutual interaction between accounts have an impact on the relationship. To subject activities to the algorithms, we collected up to 3,200 recent tweets in JSON files for each account in the network and then extracted the values for how many times accounts in the entire network retweet or quote another account in the same network, and counted the number of times that accounts mention each other.

In order to extract ego-networks and to assess network values (either weak-tie and close-knit), we applied multiple criteria to the edges and nodes. While many of them are used in data mining, they measure network activities rather like the ethnographic methods in Milroy (1987) but applied to the parameters available in digital social networks.

First, we use a linear combination in (1), in which we assign weights to the links in the network.

(1) $$\begin{array}{l} Edge\;weight=\left(w_{1}\;*\;retweet_{count}\right)+\left(w_{2}\;*\;quote_{count}\right)\\ \;+\left(w_{1}\;*\;mention_{count}\right) \end{array}$$

Where *w*_1, *w*_2, and *w*_3 are weights that can be assigned based on the application of interest so that $\overset{3}{\underset{i=1}{\sum }}w_{i}=1.$ Weights regulate the importance of each feature in the analysis. For instance, if we want to focus on the number of retweets, we assign *w*_1 = 1 while *w*_2, *w*_3 = 0. Moreover, we assume that those accounts that have a higher rate of publishing tweets have more impact on the information flow in a network, which should be considered as a factor. The point is to separate active accounts from those that use Twitter passively while rarely creating any content. To assign weights, we extracted the age (in days) of each account and the total number of tweets. Then, calculating the average number of tweets per day for each account and using (2), we can assign weights to the individual nodes as well.

(2) $$\begin{array}{l} Node\;weight\;\left(A\right)=\;\frac{average\;tweets\;per\;day\;for\;account\;A}{W}, \end{array}$$

(3) $$\begin{array}{l} where:\;W=\;\sum_{i=1}^{N}average\;tweets\;per\;day\;for\;account\;A_{i}. \end{array}$$

**Figure 7:**
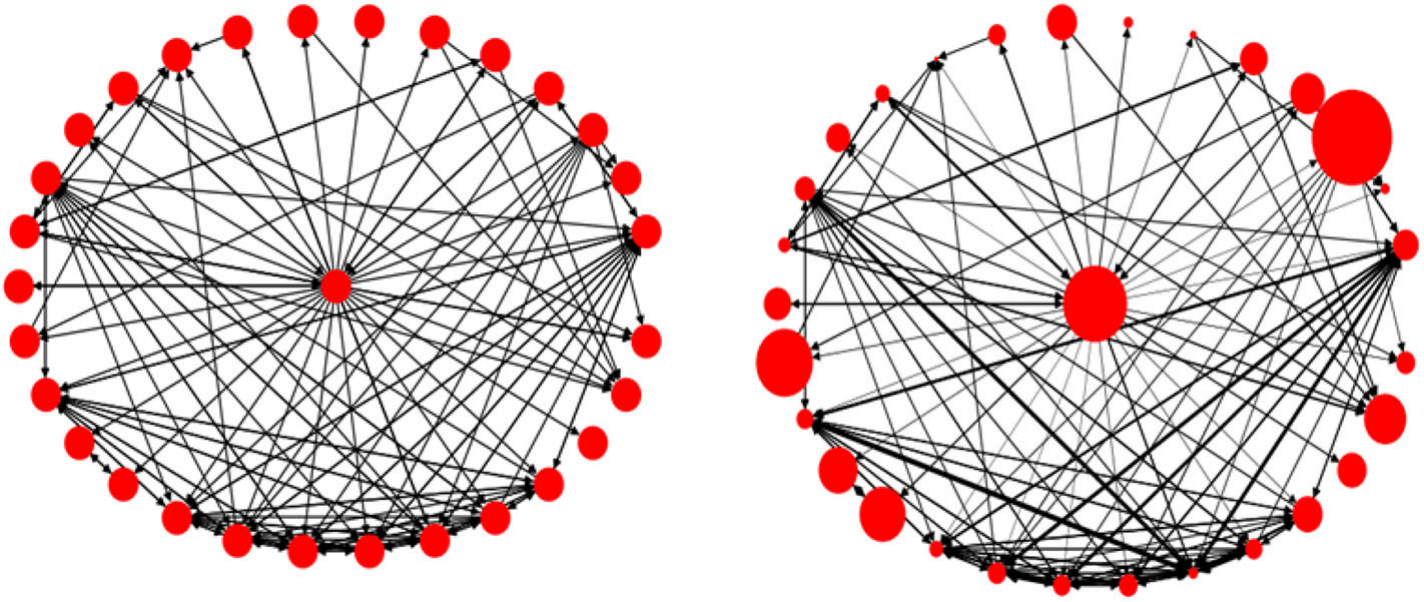
An ego network, without assigning weights (**Left**), and with weights (**Right**).

Figure 7 visualizes an ego network with 30 nodes and 142 edges, (a) without assigning weights to the nodes and edges, and (b) by assigning weights using the formulae in (1)–(3). The larger the node, the higher the value for tweets per day, and the thicker the link, the stronger the connection between the nodes.

Second, we use *betweenness centrality* (BC) to detect the density and to interpret how people in a network are connected to each other. The BC values represent the ratio with which an account establishes the shortest path between any pair in the network (Freeman, 1977). In other words, the BC of node *v* is the sum of the fraction of all of the shortest paths for any pair of nodes in the network that pass through *v*:

(4) $$\begin{array}{l} C_{B}\left(v\right)=\sum_{s,t\;\in \;V}\frac{\sigma \left(s,t|v\right)}{\sigma \left(s,t\right)} \end{array}$$

Where *V* is the set of nodes in the network, σ(*s, t*) is the number of shortest paths between nodes *s* and *t*, and σ(*s, t*|*v*) is the number of shortest paths between *s* and *t* that pass through *V*. Hence, the lower the BC value, the fewer the shortest paths passing through that account, and *vice versa*. The assumption is that the lower the spread (i.e., the difference between the higher and the lower values) of BC values in a network, the more connected the accounts are to each other, and the network consists of strong ties.

**Figure 8:**
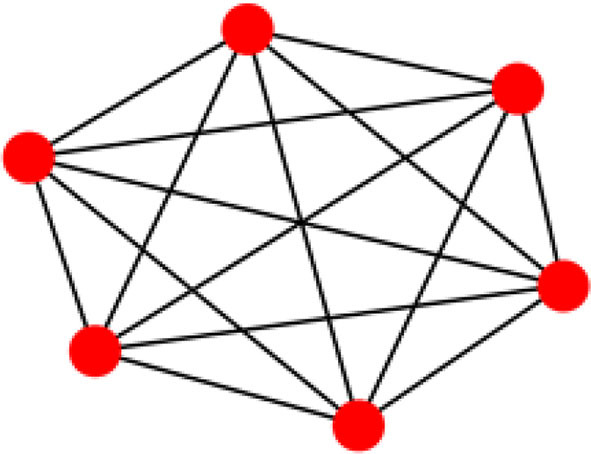
A complete graph with 6 nodes and BC mean and spread.

Consider Figure 8, in which the spread of the BC values is zero. The network is complete as all the nodes are connected to each other and the shortest path between each pair of the nodes is the direct path between those two nodes, and the path does not pass through any other nodes.

**Figure 9:**
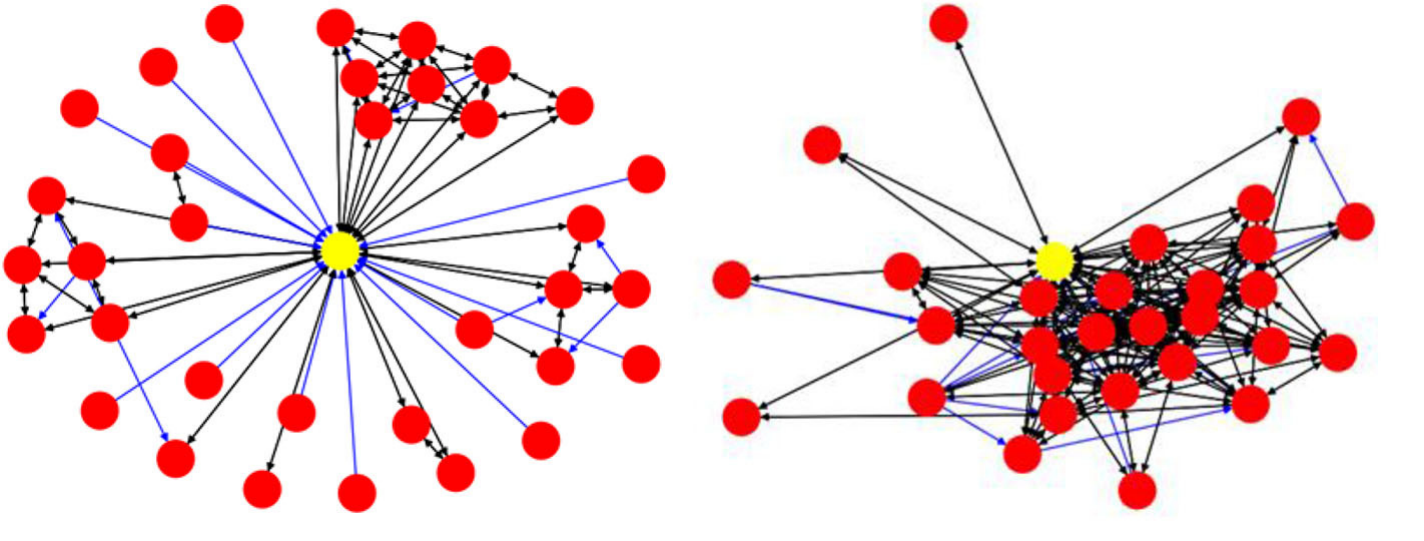
A weak-tie ego network (**Left**) and a strong-tie network (**Right**).

Figure 9 visualizes two real Twitter networks. The yellow nodes represent the ego, while the black links represent Two-way connections and blue links show One-way connections. Using visual cues, we can see that the left side is a weak-tie network, while the one on the right represents a stronger-tie network, and this is also supported by quantitative evidence. The spread value for the weak-tie network is 0.5455 and the corresponding value for the strong ties is 0.3014. We use normalized BC values to address the effect of network sizes on the calculations.

The third measure is *closeness centrality* (CC), a concept that measures the distance between nodes (Perez and Germon, 2016). In the graph theory, the distance between two nodes is defined as the length of the shortest path between two nodes. CC is the reciprocal of the sum of the distances from a node to all the other nodes in the network. As in the case of the BC analysis, to eliminate the effect of network size we applied the normalized CC values in the analysis. The normalized CC value is calculated using the formula in (5):

(5) $$\begin{array}{l} C_{C}\left(v\right)=\frac{N-1}{\sum_{i=1}^{N-1}d\left(u,v\right)}. \end{array}$$

Here, *d*(*u, v*) is the shortest-path distance between *u* and *v*, and *N* is the number of nodes in the network. The CC values are between 0 and 1 for each node, and higher values of closeness on average could be interpreted as higher connection rates between nodes. In a directed graph in Twitter, there are two CC values for each node (i.e., incoming and outward). If the difference between the two CC values on average is low, it indicates that the majority of the connections in a network are Two-way links. Therefore, the network is a stronger-tie network.

The next two measures have been purpose-built by us and can be illustrated by inspecting the two networks in Figure 9, above. In the weak-tie network (left), the majority of the accounts are connected to each other through the ego node, while the accounts in the right-hand network are not only connected to the ego node but to the other accounts in the network as well, which means that the network consists of stronger ties. If we remove the ego node and its incoming/outgoing links from the data, we can then calculate the ratio of *disjoint nodes* in the network. We assume that the higher the value of the disjointness ratio, the weaker the network will be. Furthermore, as mentioned before concerning the edge weights, we can calculate the mean values of the edge weights for each network. We would argue that, for a stronger-tie network, the mean value of the edge weights should be higher than the corresponding value for a weaker-tie network because individuals in a strong-tie network might be expected to have more interaction and activities than in a weaker-tie network.

The last algorithm strengthens the method by bringing in a tool that enables us to measure the similarity between two sets. It builds on the idea that individuals in a strong-tie network might be expected to be more similar to each other than individuals in a network characterized by weak ties. If we use Milroy's (1987) ethnographic work as our point of comparison, men in the Belfast neighborhoods were localized and spent more time with those who were similar to themselves in their dense strong-tie networks than women.

To measure similarity between sets, we use the Jaccard Similarity Coefficient (JSC). It is a symmetric measure that can be used to calculate the similarity between sets A and B as follows:

(6) $$\begin{array}{l} JSC\;=\;\frac{\left|A\cap B\right|}{\left|A\cup B\right|} \end{array}$$

The assumption is that if two accounts have a high number of shared friends (i.e., a high JCS value), they are more similar to each other than two other accounts with a lower JCS value. Consequently, if the average JCS values for all the nodes in ego network A are higher than the averages for another network B, it means that the accounts in the A network are more similar to each other and that we are dealing with a stronger-tie network, and *vice versa*.

Consider the two networks presented in Figure 9, above. Using the formula presented in (6), we can calculate the mean JSC value for the weak-tie network to be 0.12 and the corresponding value for the stronger-tie to be 0.9. The average similarity for the network on the right is almost 8 times higher than the average similarity for the network on the left.

**Figure 10:**
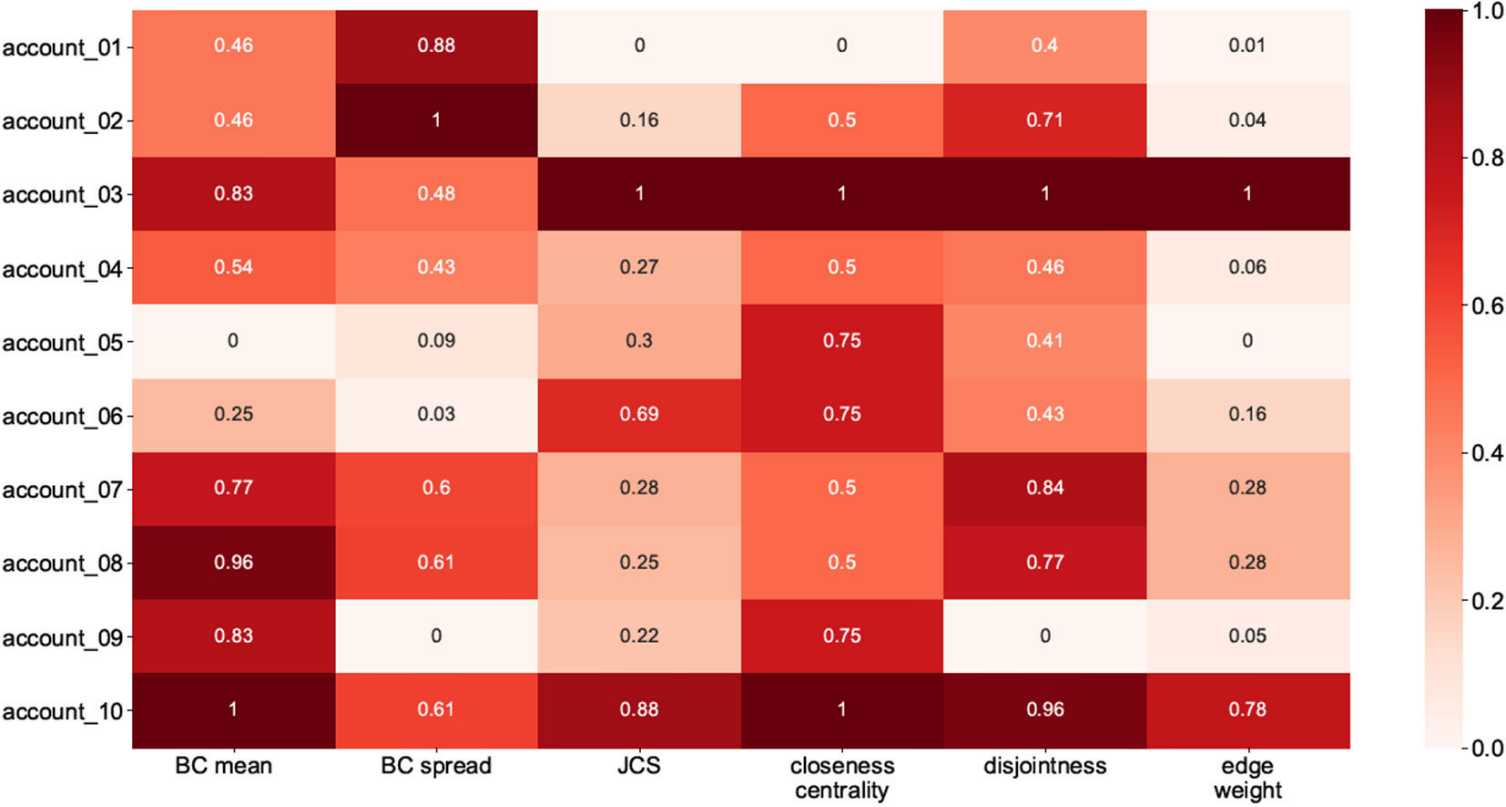
Ten candidate accounts and their corresponding values for indices.

To measure the network qualities, we extracted the values for each network and, with the aid of Min-Max normalization, placed them on an interval [0,1]. We subtracted the calculated values for the BC mean, BC spread, disjointness ratio, and CC difference from 1 in order to make them comparable with the other features. The values are shown in Figure 10. The higher values for each feature (i.e., the darker the cell) indicate stronger-tie networks, and *vice versa*.

**Figure 11:**
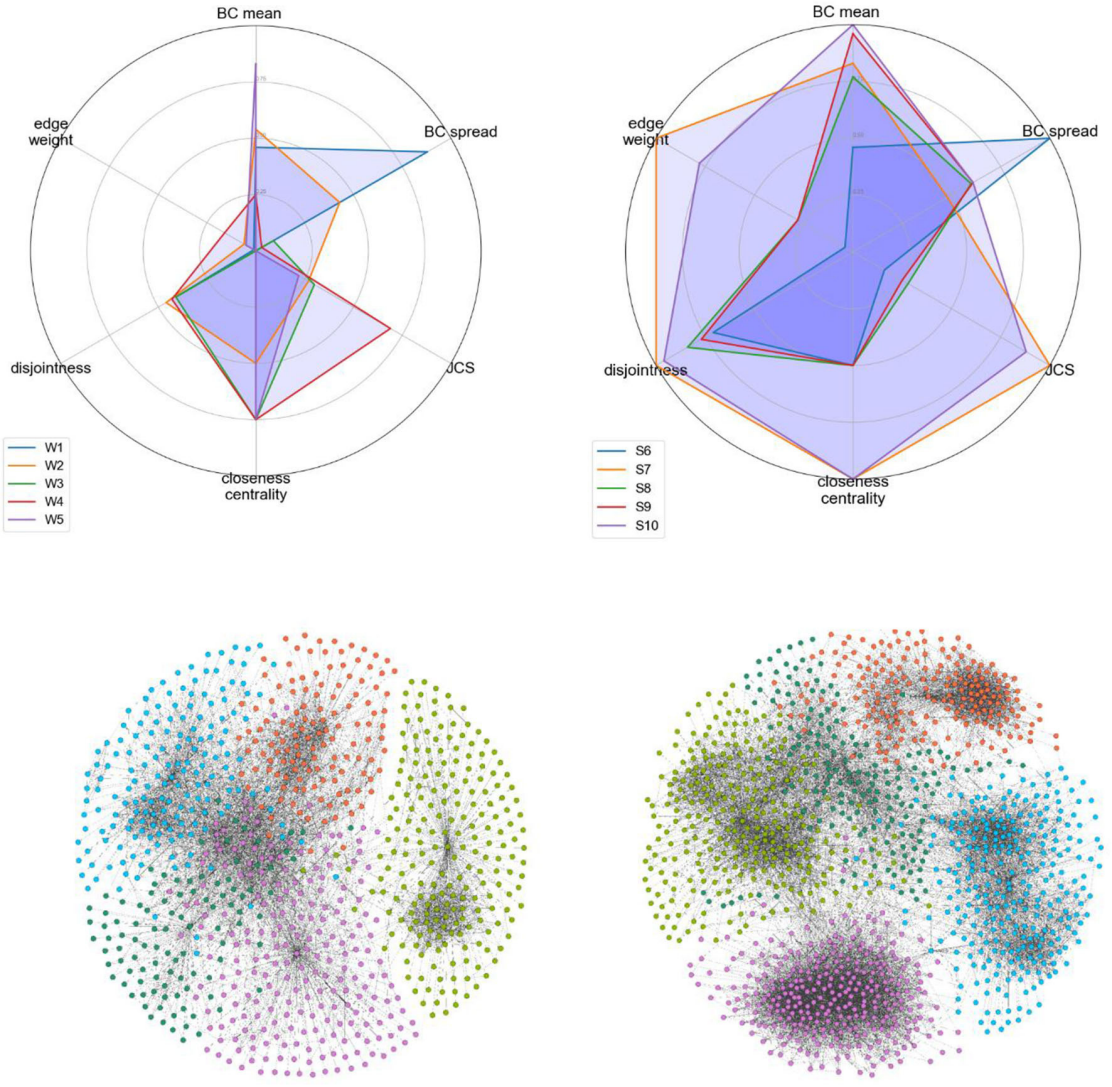
Visualizing all the candidate networks.

To assign labels (weak-tie or strong-tie) to the candidate networks, we calculated the mean values (strength coefficient *alpha*) for each cell in Figure 10. We then labeled the accounts with lower alpha values as weak-tie networks (W1–5) and the rest as strong-tie networks (S6–10), as shown in Figure 11.

The strength values (top) and the visualizations of all of the ten networks suggest that the algorithms are able to distinguish between networks with differing qualities. The visualization shows that the candidate networks as a whole can be roughly divided into weak-tie networks and networks with stronger ties. The method is robust and is not affected by smaller clusters that might appear, for instance, inside a weak-tie network. As a whole, therefore, we are able to suggest that the differences between the network types are supported by complex multidimensional quantitative data and visual cues. The next step is, then, to test to see whether differing network structures are reflected in the linguistic behavior.

In the last part of this study we investigate how the dependent variables, listed in section Materials and Methods above, are distributed among the network types. The accounts, their sizes, and the normalized frequencies (per 100,000 messages) of the dependent variables are shown in Table 2, below. The three columns on the right show the number of English messages in the network, the number of contractions in the text, and the frequency of *NEED to* + V constructions. It is important to note that, while the observations are based on a limited number of accounts, the data have been retrieved from the entire network connected to the ego node. These data consist of a total of 2,074 network nodes with over 2.6 million messages and nearly 30 million tokens of text. The network sizes vary, with the smallest possessing 105 nodes and the largest nearly 300. The number of messages varies between 100,915 and over half a million. The mean is 264,351 messages.

**Table 2:** Statistics related to the dependent variables (normalized per 100,000).

**Account**	**Network**	**N msg**.	**EngShare**	**Contr**.	***NEED to* + V**
W1	221	312,350	63,220	3,860	630
W2	175	150,774	40,910	940	310
W3	105	100,915	81,195	3,580	770
W4	132	192,944	45,237	3,230	560
W5	216	189,628	68,688	3,800	610
S6	166	253,758	79,534	2,590	840
S7	195	286,945	61,039	2,930	660
S8	281	316,944	85,387	5,790	890
S9	286	322,566	62,170	2,610	450
S10	297	516,686	81,261	6,290	1,070

**Figure 12:**
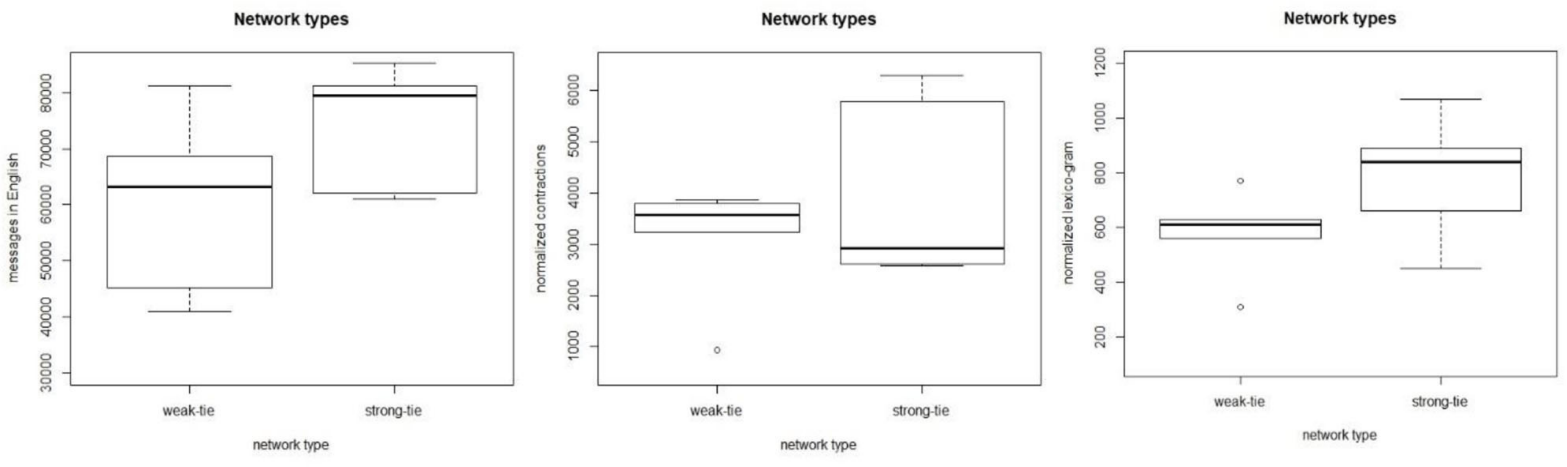
The relationships between the network types and the dependent variables.

Figure 12 shows three boxplots that visualize the relationships between the weak- and strong-tie networks and the three dependent variables. The data show no consistent pattern in which large networks would be quantitatively different from each other, but large weak and strong-tie networks behave similarly in terms of these variables. For the count of English messages (left), the mean value for the strong-tie networks is higher, but when tested with the Welch Two Sample *t*-test for independent samples, the differences between the networks are not statistically significant (*t* = −1.55, *p* > 0.05). The mean value for the contracted forms is slightly higher for the weak-tie networks, but the differences are not statistically significant (*t* = −0.97, *p* > 0.05). As for the lexico-grammatical variable, the mean is higher for the strong-tie networks, but the differences are not statistically significant (*t* = −1.55, *p* > 0.05).

The quantitative patterns observed are clear. When we investigate the large networks whose sizes are above the threshold level suggested in section A Cohort-Based Approach to Network Size, we can observe identical patterns. The results show no distinction between large weak-tie and strong-tie networks, which suggests that the differences observed in small ethnographic studies level out when the network size becomes sufficiently large. These observations support the cohort-based findings in section A Cohort-Based Approach to Network Size, above, and they also introduce ways of measuring the digital networks of mobile individuals in the social media.

We have attempted to demonstrate our algorithmic method which utilizes data-mining of the social media and uses a range of quantitative measures to establish network indices. The method enables us to establish networks of varying strengths and to determine that these varying qualities can not only be visually confirmed (Figure 11) but also supported by quantitative information. The method requires some computational power but still involves a qualitative element, since we have endeavored to ensure that the candidate networks represent similar content profiles. As we point out above, previous studies have suggested that various subpopulations have anomalously high network profiles (McCarty et al., 2001), and, at this stage, the objective has been to ensure that the candidate networks are similar. Our future objective is to test the algorithmic method with a far larger set of networks.

## Conclusions

This article has investigated digital social networks of highly mobile individuals, and we have attempted to contribute to the study of social networks in sociolinguistics by providing tools for accessing large networks. The research objective has focused on the role played by network size as a key determinant in social networks. We have shown that network size has not been used in variationist sociolinguistics. Recent network studies in other fields have, however, suggested that network size could play an important role and that the distinction between network types might level out beyond a given threshold size of networks (Ma et al., 2019). Another of our motivations has been to observe real networks whose size is close to the average (at least in Western societies). The mean size of the ego networks (207 nodes) used in section An Algorithmic Approach to Networks in Sociolinguistics far exceeds the size of networks that have been covered in previous sociolinguistic studies, but they still fall within the limits of viable networks, as discussed in section Social Networks in Variationist Sociolinguistics.

As for the research questions, the first question focused on improving the methods used in sociolinguistics so that the quantitative variable of a network could be better operationalized in situations where the population consists of both socially and geographically highly mobile individuals. We have introduced two methods for accessing the networks of mobile individuals, thus expanding the empirical basis from small-scale ethnographic observations. Section A Cohort-Based Approach to Network Size introduced cohort-based methods, while in section An Algorithmic Approach to Networks in Sociolinguistics we detailed an algorithmic approach. The methods have a strong empirical basis, and they offer new tools for variationist sociolinguistics. They reveal fundamental differences in comparison with ethnographic approaches. For instance, one of the advantages of ethnographic social network studies is that the methods build on the idea that networks are intrinsically a participant-related concept rather than something than an outsider analyst could construct (Milroy and Llamas, 2013). Our cohort-based method adopts an alternative approach, a clearly analyst-driven approach aimed at uncovering broad quantitative patterns in data rather than looking at existing networks. However, the algorithmic approach is very similar to the original idea, since the starting point is an existing network. As in Milroy and Milroy (1978) and Milroy (1987), the second method assumes the unit of study to be essentially a pre-existing category. Moreover, our method assumes network ties to be multidimensional, as the algorithms account for not only frequency of communication, but also a range of other factors. This means modernizing the network concept in sociolinguistics and bringing it closer to the contemporary idea that networks are not based on a simple dichotomy but consist of a range of attributes (Brashears and Quintane, 2018).

The second research question concentrates on the effect of network size on the validity of the theory by combining methods from sociolinguistics with computer science. Our results gained from both methods suggest that network size plays a role, and that the distinction between weak ties and stronger ties levels out once the network size grows beyond roughly 120 nodes. This finding is similar to the finding related to trust in networks (see section Social Networks in Variationist Sociolinguistics, above). We would, therefore, suggest that further studies be made of the digital networks of mobile individuals. Our raw data and the code are publicly available to other researchers.

Our future plans include continuing to work using the two methods. We plan to expand the cohort-based method and to test it with other dependent variables than simply language choice. Moreover, the metadata available in the tweet stream contain a number of possible predictors other than network size, and they need to be tested using linear regression. As for the algorithmic approach, our objective is to collect data from (tens of) thousands of accounts to scale up the method.

## Data Availability Statement

The Twitter dataset used in section A Cohort-Based Approach to Network Size is publicly available through the streaming API (https://developer.twitter.com). The data used in section An Algorithmic Approach to Networks in Sociolinguistics can be made available by the authors, without any undue restrictions, to qualified researchers. The code used in the algorithmic approach is available through GitHub (https://github.com/Masoud-Fatemi/Two-approaches-to-digital-social-networks).

## Author Contributions

This study was conceptualized by ML and MF. JL was responsible for data curation together with MF. The investigations were carried out by ML and MF. The methodology developed by MF, JL, and ML. The visualizations were created by MF and ML. The project was administered by ML, who was also responsible for writing the original draft version. All authors contributed to the article and approved the submitted version.

## Conflict of Interest

The authors declare that the research was conducted in the absence of any commercial or financial relationships that could be construed as a potential conflict of interest.
